# A Framework of Analysis to Facilitate the Harmonization of Multicenter Radiomic Features in Prostate Cancer

**DOI:** 10.3390/jcm12010140

**Published:** 2022-12-24

**Authors:** Rossana Castaldo, Valentina Brancato, Carlo Cavaliere, Francesco Trama, Ester Illiano, Elisabetta Costantini, Alfonso Ragozzino, Marco Salvatore, Emanuele Nicolai, Monica Franzese

**Affiliations:** 1 IRCCS SYNLAB SDN, Via E. Gianturco, 113, 80143 Naples, Italy; 2 Adrology and Urogynecological Clinic, Santa Maria Terni Hospital, University of Perugia, 05100 Terni, Italy

**Keywords:** MRI, radiomics, batch effects, prostate cancer, PCA

## Abstract

Pooling radiomic features coming from different centers in a statistical framework is challenging due to the variability in scanner models, acquisition protocols, and reconstruction settings. To remove technical variability, commonly called batch effects, different statistical harmonization strategies have been widely used in genomics but less considered in radiomics. The aim of this work was to develop a framework of analysis to facilitate the harmonization of multicenter radiomic features extracted from prostate T2-weighted magnetic resonance imaging (MRI) and to improve the power of radiomics for prostate cancer (PCa) management in order to develop robust non-invasive biomarkers translating into clinical practice. To remove technical variability and correct for batch effects, we investigated four different statistical methods (ComBat, SVA, Arsynseq, and mixed effect). The proposed approaches were evaluated using a dataset of 210 prostate cancer (PCa) patients from two centers. The impacts of the different statistical approaches were evaluated by principal component analysis and classification methods (LogitBoost, random forest, K-nearest neighbors, and decision tree). The ComBat method outperformed all other methods by achieving 70% accuracy and 78% AUC with the random forest method to automatically classify patients affected by PCa. The proposed statistical framework enabled us to define and develop a standardized pipeline of analysis to harmonize multicenter T2W radiomic features, yielding great promise to support PCa clinical practice.

## 1. Introduction

Radiomics is a quantitative approach to medical imaging that aims at enhancing the data available to clinicians by means of advanced mathematical analysis [1,2]. The key advantage of radiomics is the automatic extraction of high-dimensional quantitative features from medical images containing information on tumor pathophysiology. These features can be used later to facilitate clinical decision making by means of building predictive models for diagnosis and prognosis purposes [3]. 

In particular, the rapid development of medical imaging techniques and modalities has demonstrated great value in the screening, diagnosis, treatment response measurement, and prognosis evaluation of prostate cancer (PCa) [4]. PCa is the second most diagnosed cancer worldwide in men [5]. In fact, radiomic investigation has been intensively used for PCa detection, localization, staging, aggressiveness assessment, treatment decision-making assistance, and PCa patient follow-up. Recent studies have demonstrated that multiparametric magnetic resonance imaging (mpMRI) could be a better radiomic biomarker than systematic TRUS biopsy to investigate suspected PCa patients [6]. Several studies have been published so far on the diagnostic and prognostic roles of radiomics for PCa [7]. 

The increased interest in the application of radiomics in the clinical field has brought the need to carry out large multicenter studies and consequently to establish transparency in radiomics-based models. As a result, various initiatives have acknowledged the need for radiomics standardization to increase repeatability and to bring radiomics research into clinical practice [8]. Among them, the Image Biomarker Standardization Initiative (IBSI) aims to address the difficulty in reproducing and validating radiomics. In fact, standardized definitions of radiomics features with verifiable reference values are lacking, and the image processing schemes required to compute features are not implemented consistently. Moreover, the reporting in several studies is insufficiently detailed to reproduce findings [9]. According to a recent review [10], only few studies referred to the IBSI guidelines or used software for radiomic features extraction that was IBSI-compliant (e.g., PyRadiomics). A standardized methodology for extracting and processing the features is required because many institutions employ various imaging methodologies and tools, which might directly affect radiomic characteristics and delay the conversion of radiomic results into clinical practice. The effect of feature normalization choice on the reproducibility of results is an important and frequently undervalued part of the radiomic framework of analysis. Normalization standards are needed for quantitative radiomic features to reduce within-bias effects and between-bias effects. The first are associated with differences in scale, range, and statistical distributions, while the second are called batch effects and are associated with technical variations induced by different scanners or protocols and samples labeled at different times. Given the above, there is an unmet need to establish robust pre- or post-image-acquisition methods for radiomics data harmonization. There are some image-acquisition factors that generate radiomics variability. These variability-causing factors are the “batch effects” [11]. It is well known that high degrees of skewness in untransformed features can artificially lower *p*-values in statistical analysis [12]. Additionally, failing to normalize features and using incorrect normalizing techniques during post-processing steps may cause individual features to be over-represented or under-represented in statistical analyses, which could eventually introduce bias into generated models. Standards for normalizing quantitative radiomic characteristics appear to be lacking in the extant literature. On the other hand, several efforts have been shown to improve normalization procedures for image intensity values as a crucial pre-processing step. In fact, before extracting quantitative radiomic characteristics, image variability and normalization stages are crucial correction methods for imaging-related batch effects [13,14,15,16,17,18]. Different techniques were presented in a recent review [18], such as the standardization of image acquisition, the post-processing of raw sensor-level image data, data augmentation techniques, and style transfer.

Moreover, in a multicenter study, despite the normalization step, the radiomics data were affected by unwanted variation, i.e., batch effects, caused by image acquisition [19]. In this case, batch-removal statistical approaches are necessary to harmonize the data from all different batches into the same space, removing the non-biological effects. Recent studies [3,20] have shown that the choice of normalization approach influences quantitative radiomic analysis in breast cancer. Methods such as scaling, z-score, robust z-score, and upper-quartile normalization were less harsh than log-transformation, quantile, and whitening. However, care should be taken when applying a log-transformation to radiomic data since it is possible to exacerbate rather than decrease the skewness of the distribution. On the other hand, the quantile normalization method transforms original data to remove undesirable technical variation. Although this method has proved to be effective in practice, it has the danger of removing important data and artificially introducing features that are hardly distinguishable between samples [21]. The use of whitening normalization with PCA can make a more substantial normalization of the features to give it zero mean and unit covariance, resulting in decorrelated transformed features [22]. However, this transformation has the drawback of stretching all input dimensions (including noise) to the same size, which can substantially amplify data noise. 

Additionally, the majority of radiomics investigations have been retrospective single-center studies, and the majority of published models did not include external validation. In fact, one of the most challenging aspects is related to the different characteristics of medical images when acquired on different scanner models from different manufacturers using different acquisition protocols and reconstruction settings, which is currently an unavoidable scenario in clinical practice. It has been demonstrated that radiomic characteristics are sensitive to this heterogeneity, which makes it difficult to pool data for statistical analysis and/or machine learning (ML) in order to create reliable models. Recent reviews [18,19,23] discussed existing methods to perform data integration and harmonization with the aid of reducing the unwanted variation associated with batch effects. Different techniques of removing batch effects have been widely used when combining data from different experiments consisting of two or more microarray gene expression datasets exploited in a downstream analysis. The different batch effect removal methods can be categorized into three groups: location-scale (LS), matrix factorization (MF), and discretization methods, which include methods such as the identification of reproducible feature intensity harmonization, ComBat and its derivatives, and normalization using deep learning [18].

Less interest has been shown in radiomics. However, the most used methods to remove the batch effects in radiomics are the ComBat method and its variations [11,18,24,25,26]. One study by Ibrahim et al. [27] presented a framework involving a robust radiomics analysis and the application of a post-reconstruction feature harmonization method using ComBat to remove batches affecting radiomic features. A similar framework was used in another study [28] to assess the performance of ComBat on a CT phantom dataset obtained with the same acquisition and reconstruction parameters, except for the in-plane resolution, on two CT scanner models.

Only a few studies investigated a method other than ComBat, for instance, singular value decomposition and voxel size [11].

Therefore, this study aimed to develop a statistical framework of analysis to facilitate the harmonization of multicenter radiomic features to develop robust non-invasive biomarkers to translate to clinical practice by investigating different batch effect removal methods that are widely used in genomics but less considered in radiomics. The rationale behind this study was also to investigate which batch effect methods are more effective for removing the center effect while retaining the pathophysiological information in order to facilitate multicenter studies and the exportation of a radiomic model to different centers.

This framework was developed considering T2W MR images due to the high spatial resolution and the superior anatomical details they provide [29,30].

## 2. Materials and Methods

### 2.1. Datasets

#### 2.1.1. Center 1

Patient imaging and histopathology records were collected at H.S. Maria delle Grazie, Italy. Prior to the MR examination, informed consent was acquired. This retrospective study comprised 152 individuals who received prostate MRI between April 2013 and September 2018 due to increased PSA levels and/or the clinical suspicion of PCa and, subsequently, biopsy. Further information can be found in Brancato et al. [31]. The study was conducted in accordance with the Declaration of Helsinki, and the study protocol was approved by the ethics committee of the Istituto Nazionale Tumouri “Fondazione G. Pascale” (protocol number 1/20). Biopsy results were considered the gold standard. In particular, lesions were classified as positive for PCa in cases of GS ≥ 3 + 3.

#### 2.1.2. Center 2

Patient imaging and histopathology records were collected at H.S. Maria di Terni, Italy. Informed consent was obtained before MR examination. In total, 58 patients who underwent MRI of the prostate between July 2015 and October 2021 due to elevated PSA levels, and/or the clinical suspicion of PCa and, subsequently, biopsy were included in this retrospective study. The study was conducted in accordance with the Declaration of Helsinki, and the study protocol was approved by the Regional Ethics Committee of Umbria (protocol number 25069/22/ON). Biopsy results were considered the gold standard (PCa-positive lesions in cases of GS ≥ 3 + 3).

### 2.2. Image Acquisition

For center 1, the MRI acquisition protocol included T2W, T1W, DCE-MRI, and DWI (b values of 50, 400, and 1000 s/mm^2^) images. The DWI included an apparent diffusion coefficient (ADC) map generated at the time of acquisition. Patients were injected with the contrast agent Gadoteridol (Gd-HP-DO3A; Pro Hance, Bracco Diagnostics, Princeton, NJ, USA) at a dose of 0.1 mL/kg before DCE-MRI acquisition. All patients were imaged using a MAGNETOM-Avanto scanner (Siemens Healthcare, Erlangen, Germany) at 1.5 T with both an endorectal coil and a phase-array pelvic coil. For center 2, the MRI acquisition included T2W images, T1W images, and DWI (b value range of 0–2000 s/mm^2^) including an ADC map generated at the time of acquisition. All patients were imaged using a MAGNETOM-Verio scanner (Siemens Healthcare, Erlangen, Germany) at 3 T with both an endorectal coil and a phase-array pelvic coil. The technical parameters of the MRI sequences are detailed in the Supplementary Materials.

### 2.3. Biopsy Protocol

All prostatic biopsies were TRUS-guided and performed under anesthesia using an 18-gauge Tru-Cut needle. Each patient underwent both systematic biopsies (average of 12 random samples of the entire prostate gland) and target biopsies (at least three samples taken from each lesion identified by MRI). The number of randomly taken samples depended on the dimensions of the prostate gland, and the number of target samples depended on the dimensions of each lesion. Target sampling was carried out utilizing an MRI/TRUS fusion, alternately employing the cognitive approach or specialized software, along with ultrasound platforms from different manufacturers. The gross description included the number and core lengths of the needle biopsies. The specimens were fixed in buffered 10% formalin and routinely processed. Thin sections of four microns were cut and stained with hematoxylin and eosin (H&E). Supplementary Materials were performed for possible immunohistochemical stains to prove the loss of basal cells in small foci of cancer (p63 and high-molecular-weight keratin), combined with other antibodies that are overexpressed in prostatic cancer (anti-AMACR/p504S). One senior pathologist (with more than 10 years of experience in prostate specimen interpretation), who was blinded to the MRI reports, reviewed the pathological slices and classified the tumors according to the 4th WHO classification, further grading them using GS and the cancer group grade. The final pathology report also included the tumor extent in each needle biopsy and the percent core involvement by tumor.

### 2.4. Image Processing and Radiomics Feature Extraction

Two experienced radiologists were asked to draw 3D regions of interest (ROIs) in the biopsied lesions in consensus while also looking at the b = 1000 (for center 1) and b = 1500 (for center 2) DWI volume. Lesions were segmented utilizing in-house-developed software for region labeling. The radiologists were blinded to both the histology results and all clinical information related to the retrospective prostate MR images during the segmentation procedure. Normalization was applied to T2W image intensities prior to radiomic feature extraction. Specifically, intensities were centered at their respective means with standard deviations of all grey values in the original image [32,33,34,35]. B-spline interpolation was used to correct variability from parameters related to voxel size and to unify the voxel size across the cohort. In particular, MR images were resampled to an isotropic voxel size of 2 × 2 × 2 mm^3^ to compromise between in-plane and out-of-plane information interpolation [9,36]. As suggested by the PyRadiomics community, each image was discretized by resampling the grayscale values using a fixed bin width, allowing us to obtain an ideal bin count in the range of 16–128. Therefore, a preliminary extraction of the grayscale ranges was performed within all ROIs, and the optimal bin width of 3, which maximized the number of ROIs falling in the above-mentioned range of bins, was chosen.

A total of 1302 radiomics features were extracted from segmented ROIs using the open-source Python package PyRadiomics (https://pyradiomics.readthedocs.io/en/latest/, accessed on 1 July 2022). The extracted radiomics features comprised five groups: (1) 14 shape features; (2) 18 first-order features (intensity statistics); (3) 74 multidimensional texture features, including 23 gray-level co-occurrence matrix (GLCM), 16 gray-level size zone matrix (GLSZM), 16 gray-level run length matrix (GLRLM), 14 gray-level dependence matrix (GLDM), and 5 neighboring gray-tone difference matrix (NGTDM) features; 1196 transformed first-order and textural features, including (4) 736 wavelet features in frequency channels LHL, LLH, HHH, HLH, HLL, HHL, LHH, and LLL, where L and H are low- and high-pass filters, respectively; and (5) 460 LoG-filtered features with sigma values ranging from 1.0 to 5.0 and step size = 1. The first-order and multidimensional texture features (groups (2) and (3)) were grouped together and are referred to as “original features” The computing algorithms can be found at www.radiomics.io (accessed on 1 July 2022) and the IBSI presented a document to standardize the nomenclature and definition of radiomic features [9].

### 2.5. Techniques for Radiomics Variability Batch Correction

There are different sources of variability due to biological (between subjects) and technical effects (everything downstream from obtaining radiomic features).

Relevant sources of technical variability (batches) were identified for further correction. The main sources of unwanted variation were due to differences in acquisition parameters, acquisition protocols, site procedures, scanner configurations, imaging reconstruction techniques, image analysis, and other intrinsic features as well as unknown factors [1]. Although the usual approach in batch correction is to protect the known informative covariates and remove all remaining heterogeneity, some methods could remove unknown but important biological differences between samples. 

#### 2.5.1. ComBat

ComBat correction was applied using the SVA package (by Jeffrey T. Leek, https://bioconductor.org/packages/release/bioc/html/sva.html, accessed on 1 January 2022) from R version 3.6.1 [37]. 

The ComBat approach was first employed in genomics, but it is now commonly utilized in radiomics as well. ComBat is primarily based on an empirical Bayes framework to eliminate batch effects. The ComBat method determines a batch-specific transformation aimed at expressing all data in a common space devoid of center effects and has shown robustness with small sample sizes. In radiomics, ComBat works by first standardizing the data. Then, hyperparameters, which are used to compute the empirical Bayes estimates of conditional posterior means feature-wise by center for the center effects parameters, are estimated by the method of moments.

#### 2.5.2. SVA

SVA, among other methods, has been developed to account for the unknown/latent batch variables for high-throughput platforms [38]. This leads to the detection of features that are consistently different between groups, removing all common sources of latent variation. However, in some cases, latent variables may be important sources of biological variability [37]. This approach has previously been shown to result in more accurate and stable gene rankings, improved false discovery estimation, and correct *p*-value distributions [39]. For radiomics studies, it has not been widely used.

#### 2.5.3. ARSyNseq Method

ARSyNseq (ASCA (ANOVA-simultaneous component analysis) removal of systematic noise) is a novel strategy based on the ASCA model developed by Smilde et al. [40] to remove structural noise from microarray datasets. ASCA combines analysis of variance (ANOVA) and principal components analysis (PCA) to analyze multifactorial omics datasets. ASCA has been used for exploratory analysis and for the identification of responsive genes in transcriptomics [41]. Therefore, ARSyNseq uses the PCAs of the ANOVA parameters and residuals in the ASCA model in order to identify and distinguish noise and signals in microarray data. Following this decomposition, the relevant data elements are reconnected to reconstruct a filtered gene expression matrix that is free of structural biases. To the best of the authors’ knowledge, only genomics research has used the ARSyNseq technique.

#### 2.5.4. Mixed Effect Model

In genomics, batch effects can be modeled in mixed effects models in which all cells from each batch share a random effect. Variations of mixed effect models include the nested fixed effect models and nested mixed effect models, which were designed for scRNA-seq, and they belong to the single-gene-based methods, which ignore potential common information shared among all genes, which in turn might result in a loss of power [42].

The mixed effect model assumes the batch effect to be a random variable, and these are usually assumed to follow a normal distribution. Therefore, there is no hard assumption that the average batch effect in the given data is the same across groups, even though on the population level (when the number of batches is infinite) we assume the average to be the same [38]. To the best of the authors’ knowledge, the mixed effect model has never been used for multicenter radiomics studies. 

### 2.6. Framework of Radiomic and Statistical Analysis

**Figure 1:**
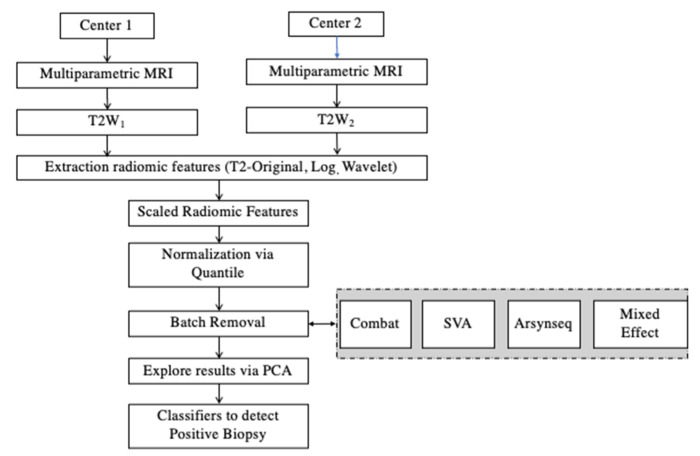
Framework of analysis. MRI: Magnetic Resonance Imaging; PCA: Principal Component Analysis; T2W = T2-weighted; SVA = Surrogate Variable Analysis.

The proposed framework of analysis is shown in Figure 1. MRI images were acquired with different acquisition protocols from centers 1 and 2. In particular, T2W images were explored and radiomic features were extracted to explore the radiomics variability and identify the sources of variability in the multicenter study. ADC images were also explored, and radiomic features were extracted, but batch effects were not visible. 

Then, image post-processing and batch correction methods were implemented to reduce the radiomics variability. First, extracted radiomic features were scaled. Each radiomic feature was centered and scaled according to the generic function in R: scale. This was performed to minimize their basic differences in scale and range [20]. Second, the scaled radiomic features were further normalized by a quantile normalization method. The original data were transformed using the quantile normalization approach. Quantile normalization, which transforms the original data to remove unwanted technical variation by forcing the observed distributions to be the same as the average distribution, which is obtained by taking the average of each quantile across samples, was used as the reference [43]. Castaldo et al. [3,20] showed that quantile normalizations outperformed other normalization techniques to characterize cancer subtypes, grades, and aggressiveness. Third, four different batch correction methods were implemented: ComBat, SVA, ARSyNseq, and a mixed effect model. Principal components analysis (PCA) was used to assess the impact of harmonization. Moreover, different classification approaches (LogitBoost, random forest, K-nearest neighbors, and decision tree) were trained, validated, and tested using the principal components (PCs) of each transformed dataset to automatically detect patients affected by PCa.

A statistical analysis and classification were performed using R software (version 3.6.1, Vienna, Austria) [44]. Continuous variables were expressed as means, standard deviations (SD), medians, and ranges. The Shapiro–Wilk test was used to determine the normality of data. Radiomics data were tested for normality before and after the application of the normalization methods. The Wilcoxon rank-sum test or t-test were used, as required, for comparisons between groups. Categorical variables were expressed as percentages and were compared using the chi-square test or Fisher’s exact test. A *p*-value less than 0.05 was considered significant. Holm’s correction was used for multiple-hypothesis correction, if necessary. Patients with multiple lesions were considered as single patients.

#### 2.6.1. PCA

A PCA was applied to radiomic features in five datasets: (1) untransformed scaled radiomic features; (2) transformed radiomic features with ComBat; (3) transformed radiomic features with SVA; (4) transformed radiomic features with ARSyNseq; and (5) transformed radiomic features with the mixed effect model. Cumulative variance was set to 60% to select the minimum number of PCs. To find the batch removal strategies that could best describe the variance in the data using PCA, we investigated PC1 and 2 of the untransformed scaled radiomic characteristics. For each dataset, the loading and variable contributions were investigated. The PCs selected for all methods were then investigated via the Wilcoxon sign rank test among positive and negative biopsies.

#### 2.6.2. Classification Approaches

On the basis of PCs that could account for 60% of the cumulative variance, classification methods were considered to automatically classify positive biopsies to PCa. These approaches were investigated in order to empirically determine the impacts of the batch effect methods. The dataset was split into 80% for training and cross-validation and 20% for testing. The rationale behind this division was that, to minimize bias and overfitting issues, a classifier should be tested on a separate set of data. We performed “stratified sampling”, where the split of training and testing was made by preserving the percentage of samples for each class (positive and negative biopsies).

Repeated (N = 100) 10-fold cross validation was employed. Since there was a ~34% rate of events (negative biopsy), we employed a synthetic minority over-sampling technique (SMOTE) to facilitate the training of the models. 

Four traditional classification methods were investigated: additive logistic regression (LogitBoost), namely a boosting algorithm that operates as an approximation to additive modeling on the logistic scale using the maximum Bernoulli likelihood; random forest trees (RF), namely an ensemble learning method for classification that operates by building a multitude of decision trees during training and outputting the class that is the mode of the classes (classification) [44]; k-nearest neighbors (KNN), which finds a group of K objects in the training set that are the closest to the test object and bases the assignment of a label on the predominance of a particular class in the neighborhood [45]; and decision trees (ctree), which are built from a set of training data using the concept of information entropy [46].

Regarding model parameters, we employed the default configuration provided in RStudio for LogitBoost and RF [47]. For LogitBoost, the number of boosting iterations was set to 100. In the random forest analysis, the number of available variables for splitting at each tree node was calculated as the square root of the number of predictor variables (rounded down). Due to the binary nature of the classification problem, K equals 1, 3, 5, 7, and 9 were used in the KNN’s training. Decision trees were created by adjusting the minimum number of instances per leaf from 2 to 20 and the confidence factor for pruning from 0.05 to 0.5.

These classification methods were chosen based on the small sample size and the existing literature. In fact, LogitBoost, decision trees, and KNN have been widely used in PCa detection [4].

To assess the impacts of the batch removal approaches for radiomic analysis, binary performances were obtained for each ML method across all methods used in this work. In addition, 95% confidence intervals (CIs) are also reported for all binary performances for the repeated 10-fold cross validation. The best-performing model was chosen as the classifier with the highest accuracy, which is a reliable estimator of both sensitivity and specificity rates; in the case of equal accuracy, the one with the highest AUC was picked. A Wilcoxon sum-rank test was used to compare the categorization algorithms over 100 repeats. The values of accuracy and AUC were also graphically investigated via boxplots. R software (version 3.6.1, Vienna, Austria) [33] was used to build the classifiers.

## 3. Results

### 3.1. Study Population

For this study, 154 PCa lesions were identified in 152 patients (102 patients with positive biopsies and 50 patients with negative biopsies) at center 1, and 58 PCa lesions were identified in 58 patients (35 patients with positive biopsies and 23 patients with negative biopsies) at center 2. The demographics and clinical characteristics of the study population are reported in Table 1.

### 3.2. Batch Effect Removal

#### 3.2.1. Radiomic Feature Characterization via PCA

PCA was applied to radiomic features in five datasets: (1) untransformed scaled radiomic features; (2) transformed radiomic features with ComBat; (3) transformed radiomic features with SVA; (4) transformed radiomic features with ARSyNseq; and (5) transformed radiomic features with a mixed effect model.

**Figure 2:**
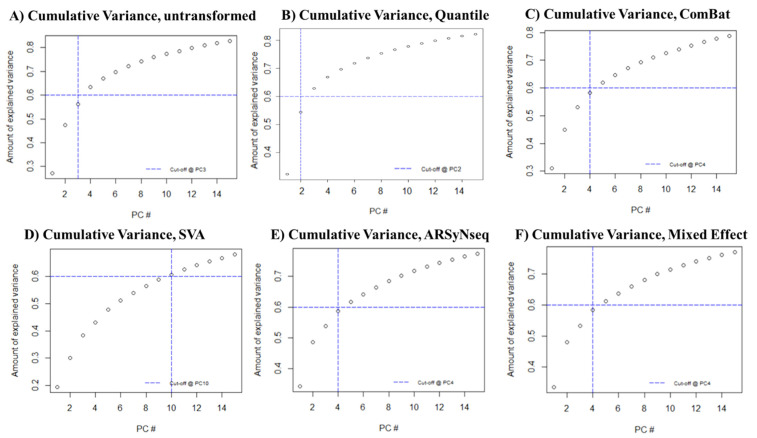
Cumulative variance plots for the 5 datasets, based on a threshold of 0.6. (**A**) Untransformed scaled; (**B**) quantile: (**C**) ComBat; (**D**) SVA; (**E**) ARSyNseq; and (**F**) mixed effect model.

The results (Figure 2) showed that 3 PCs explained 60% of the total variance in the case of untransformed radiomic features; 4 PCs explained 60% of the total variance in the case of transformed ComBat radiomic features; 10 PCs explained 60% of the total variance in the case of transformed SVA radiomic features; and 4 PCs explained 60% of the total variance in the case of transformed ARSyNseq and mixed effect model radiomic features.

The top 10 radiomic features that contributed to the PCs for each method are reported in Supplementary Figure S1.

**Figure 3:**
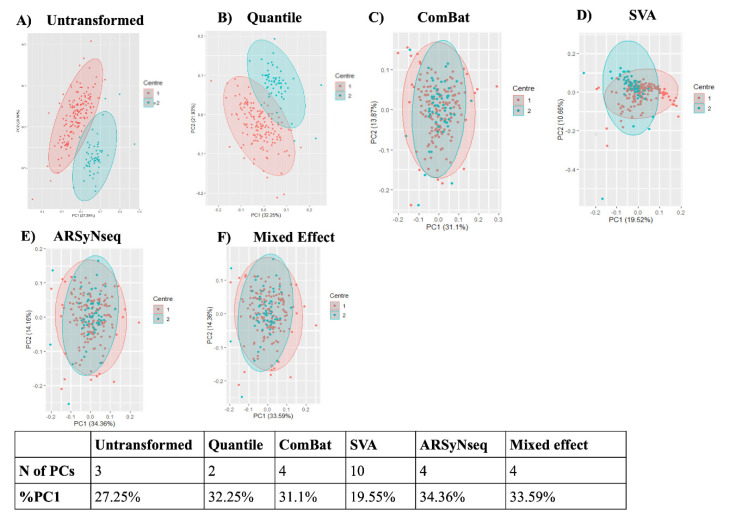
PCA scatter plots and summary table of N of PCs and percentage of variance explained by PC1 (%PC1). (**A**) Untransformed scaled; (**B**) quantile; (**C**) ComBat; (**D**) SVA; (**E**) ARSyNseq; and (**F**) mixed effect model.

Figure 3 shows the PCA scatter plots of top two principal components of the radiomic features across the two labels (centers) using untransformed data or data transformed with the four batch removal methods. 

Scatterplots of the top two principal components of PCA visually demonstrate the efficiency of ComBat, ARSyNseq, and the mixed effect model in removing the differences in radiomic features between labels while shifting the data to different locations. On the contrary, transformation by only quantile normalization (Figure 3B) was not sufficient to remove any batch effect. In the same way, SVA did not allow data from two labels to overlay each other. Moreover, as shown in the table below the graphs in Figure 2, the PC1 of SVA was lower than the untransformed PC1. This result clearly demonstrated that SVA was not removing the batch effect. Therefore, for the remaining analyses SVA was excluded. 

Statistical analyses were carried out for the PCs of each method (ComBat, ARSyNseq, and mixed effect) to investigate statically significant differences among positive (class 1) and negative (class 2) biopsies via the Wilcoxon sign rank test. 

**Figure 4:**
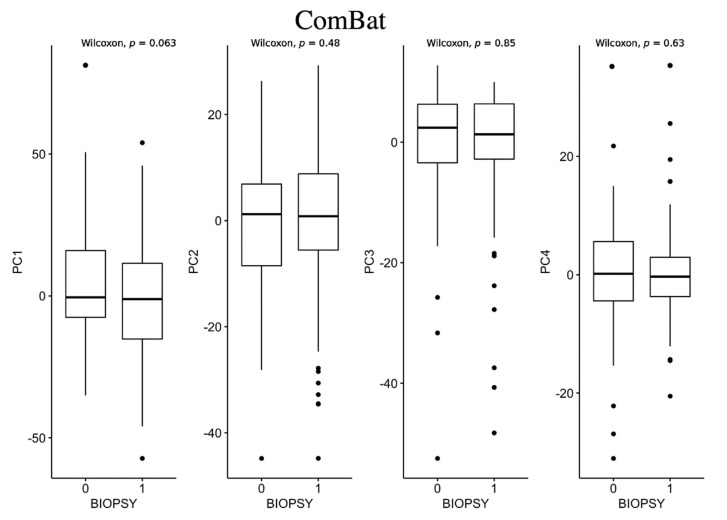
Box plots for ComBat method between negative biopsy (class 0) and positive biopsy (class 1).

As shown in Figure 4, none of the PCs computed for the ComBat method resulted in statistical differences among the two classes. Only PC3 showed a *p*-value of 0.05. 

**Figure 5:**
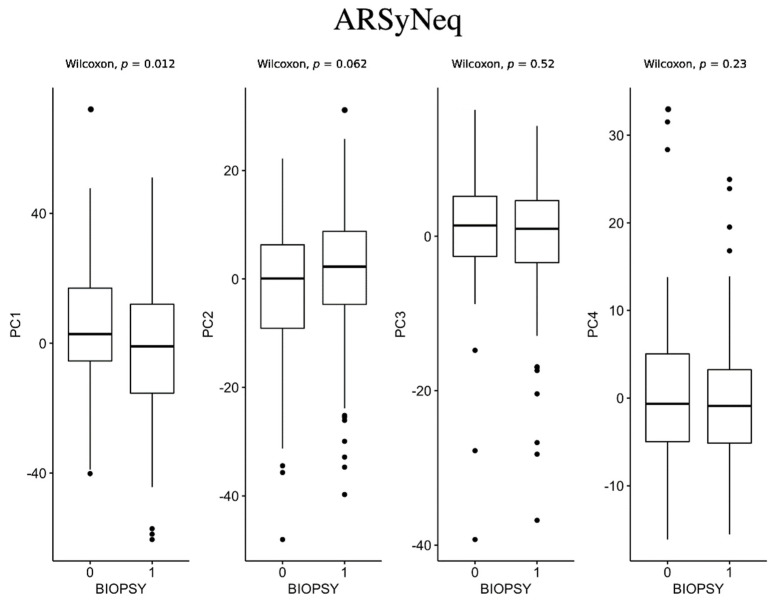
Box plots for ARSyNseq method between negative biopsy (class 0) and positive biopsy (class 1).

As shown in Figure 5, only PC1 computed for the ARSyNseq method resulted in statistical differences among the two classes. In fact, PC1 showed a significant increase (*p*-value = 0.012) in patients with negative biopsies compared to patients with positive biopsies. 

**Figure 6:**
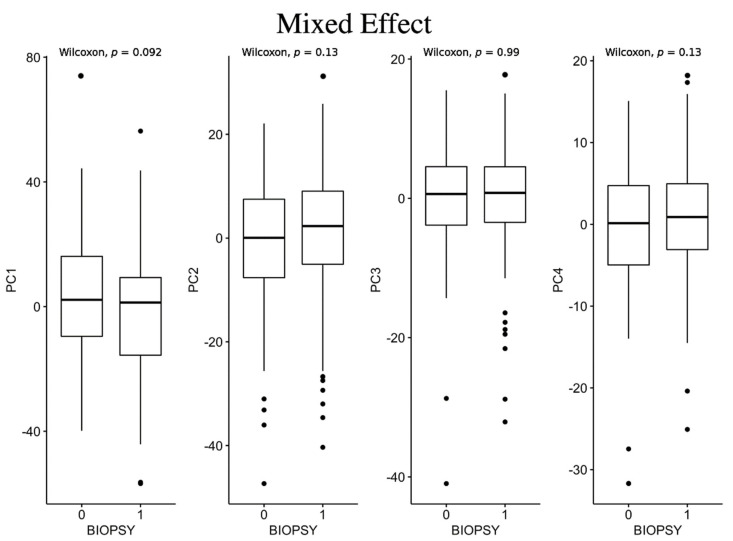
Box plots for mixed effect method between negative biopsy (class 0) and positive biopsy (class 1).

As shown in Figure 6, none of the PCs computed for the mixed effect method resulted in statistical differences among the two classes.

#### 3.2.2. PCa Patients Classification Methods

A total of 210 patients, of which 137 had positive biopsies and the remaining 73 patients had negative biopsies, were employed to train, validate, and test four classifiers by using the PCs for each method (ComBat, ARSyNseq, and the mixed effects model). The classification algorithms were trained and validated on 80% of the data and tested on the remaining 20% of the data using the first four PCs to automatically classify patients with positive biopsies (Class 1). 

**Figure 7:**
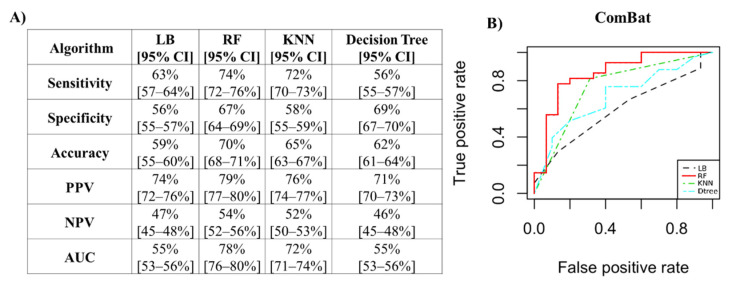
Performance measures to classify patients with positive biopsies for the ComBat method. (**A**) Algorithm performance via PCs to classify patients with positive biopsies for the ComBat method. (**B**) ROC curves of the four classifiers. PPV: positive predictive value; NPV: negative predictive value; AUC: area under the curve; LB: LogitBoost; RF: random forest; KNN: k-nearest neighbors; CI: confidence interval.

For the ComBat method, the performance measures are reported in Figure 7A. The best classifier, with an accuracy value of 70% and an AUC of 78%, was random forest, as shown in Figure 7B.

**Figure 8:**
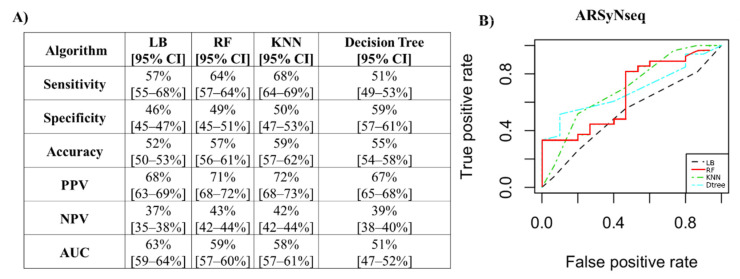
Performance measures to classify patients with positive biopsies for the ARSyNseq method. (**A**) Algorithm performance via PCs to classify patients with positive biopsies for the ARSyNseq method. (**B**) ROC curves of the four classifiers. PPV: positive predictive value; NPV: negative predictive value; AUC: area under the curve; LB: LogitBoost; RF: random forest; KNN: k-nearest neighbors; CI: confidence interval.

For the ARSyNseq method, the performance measures are reported in Figure 8A. The best classifier, with an accuracy value of 59% and an AUC of 58%, was KNN (K = 3), as shown in Figure 8B.

**Figure 9:**
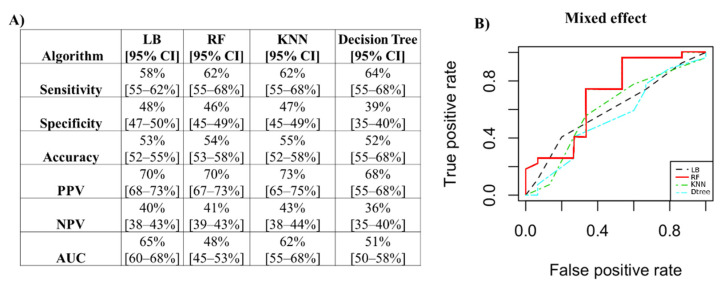
Performance measures to classify patients with positive biopsies for the mixed effect method. (**A**) Algorithm performance via PCs to classify patients with positive biopsies for the mixed effect method. (**B**) ROC curves of the four classifiers. PPV: positive predictive value; NPV: negative predictive value; AUC: area under the curve; LB: LogitBoost; RF: random forest; KNN: k-nearest neighbors; CI: confidence interval.

For the mixed effect method, the performance measures are reported in Figure 9A. The best classifier, with an accuracy value of 55% and an AUC of 62%, was KNN (K = 3), as shown in Figure 9B.

**Figure 10:**
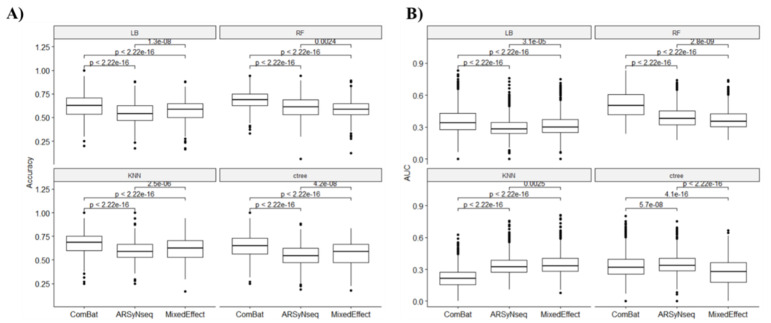
Comparison of each classifier (LB: LogitBoost, RF: Random Forest, KNN: k-nearest neighbors, ctree: decision tree) for each batch removal method used to classify patients with positive biopsies. (**A**) Boxplot of accuracy values. (**B**) Boxplot of AUC values.

As shown in Figure 10, the ComBat method outperformed the other methods (ARSyNseq and mixed effects) in terms of accuracy (Figure 10A), whereas, as shown in Figure 10B, the AUC values for the ComBat method were significantly higher for LB and the RF classifier but were lower for KNN and decision tree than for the ARSyNseq and mixed effect methods.

## 4. Discussion

This study investigated the pooling of radiomic features extracted from T2W images of PCa patients from different centers in a statistical framework. We developed a framework of analysis to facilitate the harmonization of multicenter radiomic features to develop robust biomarkers in PCa clinical practice. We investigated different normalization approaches (ComBat, SVA, mixed effect, and Arsynseq). The proposed approaches were evaluated using a dataset of 210 PCa patients from two centers and considering T2W axial images. It is worth noting that the choice of considering T2W sequences for framework development was determined by its characteristic high spatial resolution and superior anatomical details, which are crucial for the early detection and characterization of PCa [29,30]. 

ADC maps were also explored, as they have been shown to be useful for prostate cancer identification [4]. However, no batch effects were shown. Therefore, for this study we focused on T2W axial images. 

The impacts of the different normalization approaches were evaluated by PCA. The proposed framework of analysis enabled the standardization of a pipeline of analysis in order to harmonize multicenter prostate T2W radiomic features, which may lead to more precise PCa assessment. 

Variations in scanner models, reconstruction methods, and acquisition techniques are common in multicenter research and long-term retrospective investigations. Although batch effects can be reduced by careful experimental design, they cannot be eliminated unless the whole study is performed in a single batch. Moreover, combining data from different centers without carefully removing batch effects can lead to misleading results, e.g., higher ML performances. In fact, the bias introduced by the non-biological nature of the batch effects can mask or confound true biological differences. Therefore, it is necessary to identify and remove the batch effects before proceeding to the downstream analysis [48].

In this circumstance, there is an urgent need for harmonization in order to train and test efficient models. The harmonization could happen by (1) harmonizing images (i.e., pre-processing and extracting radiomic features) and (2) harmonizing features (i.e., post-processing after extracting radiomic features). The first approach relies on the standardization of acquisition protocols and reconstruction settings, which are available in existing guidelines such as the IBSI [9]. However, although following standards and guidelines may help reduce multicenter effects, it may not be enough to entirely compensate for them. Therefore, approach (2), addressing the issue in the radiomic feature domain, is still relevant. Several statistical methods exist to perform normalization or batch effect correction. The most used batch effect correction method in radiomics is ComBat. However, to the best of the authors’ knowledge, an extensive comparison of ComBat with other methods remains to be investigated. Therefore, we selected methods that were mainly used in genomics to investigate the reliability and robustness of those methods in radiomics. To compare these methods, we proposed a framework of analysis in order to reduce the batch effect in multicenter studies. 

After radiomic features were extracted from MRI images from center 1 and center 2, they were scaled and grouped in one dataset. For the first step to reduce the between-subjects bias effect, which alters the comparison of the radiomic features in different patients (namely technical effects due to their basic differences in scale, range, and statistical distributions), we applied the quantile normalization method, which was shown to outperform other methods in [3], since samples were assumed to be normalized prior to batch effect correction [4]. In fact, while normalization makes the samples more comparable, it only aligns their global patterns. Therefore, batch effects affecting the data might still represent a major source of variance, even after normalization. Thus, the diagnosis of batch effects is most informative when performed on normalized data [5]. In order to reduce the batch effect, ComBat, SVA, ARSyNseq, and mixed effect models were applied. To investigate the effects of batch effect normalization methods, PCA, which is the most common feature reduction method, was employed, and the classification methods were evaluated to investigate the patients affected by PCa. PCA was chosen as an explorative tool to visualize how different batch methods are able to disclose different aspects of the data in the scores and the accompanying loadings. Furthermore, it allows the identification of the most important radiomic features for the characterization of prostate cancer by analyzing the loadings in order to generate a combined radiomic signature [6].

PCA was applied to different datasets based on the four batch effect normalization approaches to investigate the differences among them. By investigating the cumulative variance, we could observe that the case of ComBat provided comparable results to ARSyNseq and the mixed effect model, whereas the SVA method needed 10 PCs to explain 60% of the total variance. Moreover, the scatterplots of the top two principal components of PCA visually demonstrate that SVA did not allow data from two labels to overlay each other. Moreover, PC1 of SVA was lower than the untransformed PC1. This result clearly demonstrated that SVA was not removing the batch effect. Therefore, SVA was excluded. ComBat, ARSyNseq, and mixed effect showed comparable results, identifying those methods as promising techniques for radiomics studies. 

The same radiomic features contributed to the four PCs for the ARSyNseq and mixed effect methods, whereas different radiomic features contributed to the PCs for the ComBat method. This stresses that, while working with quantitative radiomic characteristics, extreme care must be taken because by using different normalization approaches we can obtain different findings. 

To further investigate the roles that batch effect normalization methods have on the PCA-based framework, we investigated whether the PCs were able to differentiate among patients with PCa via classification approaches. The main aim of using the classification methods was to investigate the impacts of the batch effect normalization methods and the use of PCA on classification performances.

In terms of accuracy, ComBat achieved the highest accuracy value of all classification methods by outperforming the ARSyNseq and mixed effect methods. By using PCs normalized via the ComBat effect, we were able to automatically detect, with 70% accuracy and 78% AUC, patients with PCa by using the random forest method. One possible reason for the low accuracy of the model could be the small size of the patient sample. In fact, more diverse data in the training set led to higher performance. However, these results are comparable a study by Ligero et al. [11], which showed that ComBat had the highest improvement of radiomics-based classification in both the phantom and clinical applications (K-means purity of 65.98 vs. 73.20). On the other hand, Da Ano et al. [24] achieved better results. In fact, RF provided the best classification accuracy (79–89%) with a modified version of ComBat. In accordance with other studies [11,24,26], we demonstrated that the ComBat method was more effective for removing the batch effect and automatically classifying PCa patients. Moreover, the Combat approach is suitable for small sample sizes and can remove batch effects among multiple batches. The ComBat function is able to remove both known batch effects and other potential latent sources of variation [48]. In the same way, ARSyNseq is a flexible approach for the correction of systematic biases in single omic datasets for both declared (batches) or hidden sources of technical noise [41]. On the other hand, mixed effect models correct for batch as a random effect [49].

In our study, ARSyNseq and mixed effect method based corrections improved the reproducibility of the radiomics features, although these could have suffered from overcorrection, leading to a loss of biological meaning. Overall, as a rule of thumb, when there are a large number of known or unknown potential confounders, surrogate variable adjustment may be more appropriate. Alternatively, when one or more biological groups is known to be heterogeneous and there are known batch variables, direct adjustment may be more appropriate [37]. However, one of the limitations of ComBat is that it centers the data to the overall grand mean of all samples, which results in an adjusted data matrix that is shifted to an arbitrary location that no longer coincides with the location of any of the original centers. Another disadvantage is that ComBat relies heavily on labeled data [18].

Moreover, another study [27] investigated the concordance (reproducibility) of features after ComBat harmonization, demonstrating that ComBat cannot be applied to all radiomic features but rather a percentage of features, depending on the data being harmonized. Therefore, ComBat harmonization should not be blindly performed on patient data but following the estimation of adjustment parameters on a phantom dataset [27].

Therefore, future researchers should better evaluate the best methods for their case studies. Furthermore, researchers should bear in mind that the focus of harmonization techniques must be the standardization of radiomic feature values across different imaging settings and patient populations. 

Moreover, future studies will also investigate different and consistent radiomic models based on normalization using deep learning before and after batch effect removal as well as performance improvement after batch effect removal. In fact, as outlined by a recent review [18], deep learning solutions (e.g., generative adversarial networks and neural style transfer techniques) are gaining momentum for addressing variability, especially across multicenter radiomic studies. 

We present a statistical framework combining several approaches to generate a quantitatively robust and replicable radiomic signature, which may result in more precise PCa diagnosis and prognosis and help clinicians in decision making towards personalized medicine. 

However, this study presents some limitations. The primary drawback of our investigation was the small size of the patient sample, which precluded further confirmation in a larger cohort. Our findings will thus require validation in larger cohorts. 

Another limitation of our study was that unknown biological differences such as “lesion/disease dependent characteristics” could have been removed. In fact, it has proven difficult to separate heterogeneity due to technical differences from that due to unknown biological differences. Additionally, the current batch correction methods are intended to be used each time a new dataset is created due to specific differences in sample conditions and experimental techniques. In fact, if new data have to be harmonized, then they must be added to the existing pool of data to perform correctly [18,19]. Moreover, our findings should be confirmed by using other cancer types for other imaging protocols and scanners, and the actual effect on diagnostic performance using clinical data needs to be demonstrated.

## 5. Conclusions

In conclusion, the predictive ability of the radiomic models was improved with harmonization, with ComBat providing the best results. This was observed consistently through all machine learning pipelines and performance metrics. However, all batch effect removal methods presented in this study appear promising to address the batch effects in multicenter radiomic studies and to possibly raise the statistical power of those studies.

Moreover, we proposed a framework of analysis that enables us to standardize a pipeline of analysis to harmonize multicenter prostate T2W radiomic features.

## Figures and Tables

**Table 1:** Clinical details and biopsy results on per-patient and per-lesion bases.

	Value (Patient-Based)		Value (Lesion-Based)	
	Positive Biopsy	Negative Biopsy	Positive Biopsy	Negative Biopsy
No. of patients/lesions (n (%))	137 (65.24)	73 (34.76)	139 (65.57)	73 (34.43)
Median age (y (range))	68 (52–81)	64 (54–78)	-	-
Mean PSA (ng (SD))	11.57 (11.69)	8.66 (6.17)	-	-
Mean PSA density (ng/mL (SD))	0.27 (0.33)	0.12 (0.09)	-	-
Mean prostate volume (cm (SD))	50.84 (26)	76.78 (41.65)	-	-
Prostatic zone				
PZ (n (%))	-	-	104 (49.52)	52 (24.53)
TZ (n (%))	-	-	35 (16.67)	21 (10)
Gleason score				
3 + 3 (PCa) (n (%))	-	-	63 (45.32)	-
>3 + 3 (CS-PCa) (n (%))	-	-	76 (54.68)	-

PSA = prostate-specific antigen; PCa = prostate cancer (defined as Gleason score ≥ 3 + 3); CS-PCa = clinically significant prostate cancer (defined as Gleason score ≥ 3 + 4); SD = standard deviation; PZ = peripheral zone; TZ = transition zone.

## Data Availability

Not applicable.

## Supplementary Materials

The following supporting information can be downloaded at: https://www.mdpi.com/article/10.3390/jcm12010140/s1, Figure S1: Top 10 radiomic features that contributed to the PCs. (A) Untransformed only scaled; (B) ComBat; (C) SVA; (D) ARSyNseq and (D) Mixed effect model.
