# Supplementary material for: A Framework of Analysis to Facilitate the Harmonization of Multicenter Radiomic Features in Prostate Cancer
Source: J Clin Med. 2022 Dec 24;12(1):140. doi: 10.3390/jcm12010140 (PMC9821561; doi:10.3390/jcm12010140)
Supplement: Supplementary file 1 [file jcm-12-00140-s001.zip › jcm-2052287-supplementary.pdf]

Supplementary Material

MRI acquisition details

For centre 1, the acquisition parameters T2WI included: repetition time (TR)/echo time (TE) = 4740/102 ms, flip angle = 137°, matrix size = 320 × 288, field of view (FOV) = 200 × 200 mm^2, slice thickness = 3 mm for sagittal plane acquisition; TR/ TE = 5610/102 ms, flip angle = 123°, matrix size = 320 × 310, FOV = 200 × 200 mm^2, slice thickness = 3 mm for axial plane acquisition; repetition time TR/TE = 4000/102 ms, flip angle = 138°, matrix size = 320 × 310, FOV = 200 × 200 mm^2, slice thickness = 3 mm for coronal plane acquisition. The parameters for DWI included: TR/TE = 4624/77 ms, flip angle = 90°; matrix size = 128 × 128, FOV = 250 × 250 mm^2, slice thickness = 3 mm, b = 50, 400 and 1000 s/mm^2. Volumetric Interpolated Breath-hold Examination (VIBE) sequences at variable flip angles (FAs) for T1 mapping were performed with the following parameters: TR/ TE = 5.5/2.34 ms, flip angle = (2, 5)°, matrix size = 320 × 112, FOV = 208 × 417 mm^2, slice thickness = 3.5 mm. DCE MRI was performed using T1 VIBE axial dynamic sequence after injecting 0.1 mL/kg of Gadoteridol (Gd-HP-DO3A; Pro Hance, Bracco Diagnostics, Princeton, NJ, USA) and the acquisition parameters included: TR/ TE = 5.5/2.34 ms, flip angle = 10°, matrix size = 320 × 112, FOV = 208 × 417 mm^2, slice thickness = 3.5 mm with 32 measurements on axial plane.

For centre 2, the acquisition parameters T2WI included: TR/TE = 3430-7270/89-112 ms, flip angle = 140°, matrix size = 320 × 320, FOV = 180 × 180 mm^2, slice thickness = 2-3.5 mm for sagittal plane acquisition; TR/ TE = 5000-6500/93-112 ms, flip angle = 140°, matrix size = 320 × 320, FOV = 180 × 180 mm^2, slice thickness = 3 mm for axial plane acquisition; repetition time TR/TE = 4400-6010/91-112 ms, flip angle = 140°, matrix size = 320 × 320, FOV = 180 × 180 mm^2, slice thickness = 2-3.5 mm for coronal plane acquisition. VIBE sequences for T1 mapping were performed with the following parameters: TR/ TE = 4.13/1.23 ms, flip angle = 9°, matrix size = 320 × 187, FOV = 332 × 425mm^2, slice thickness = 2-3.5 mm. The parameters for DWI included: TR/TE = 7000-1200/75-93 ms, flip angle = 90°; matrix size = 128 × 128, FOV = 250 × 250 mm^2, slice thickness = 2-3.5 mm, b-values = [0-2000] s/mm^2.

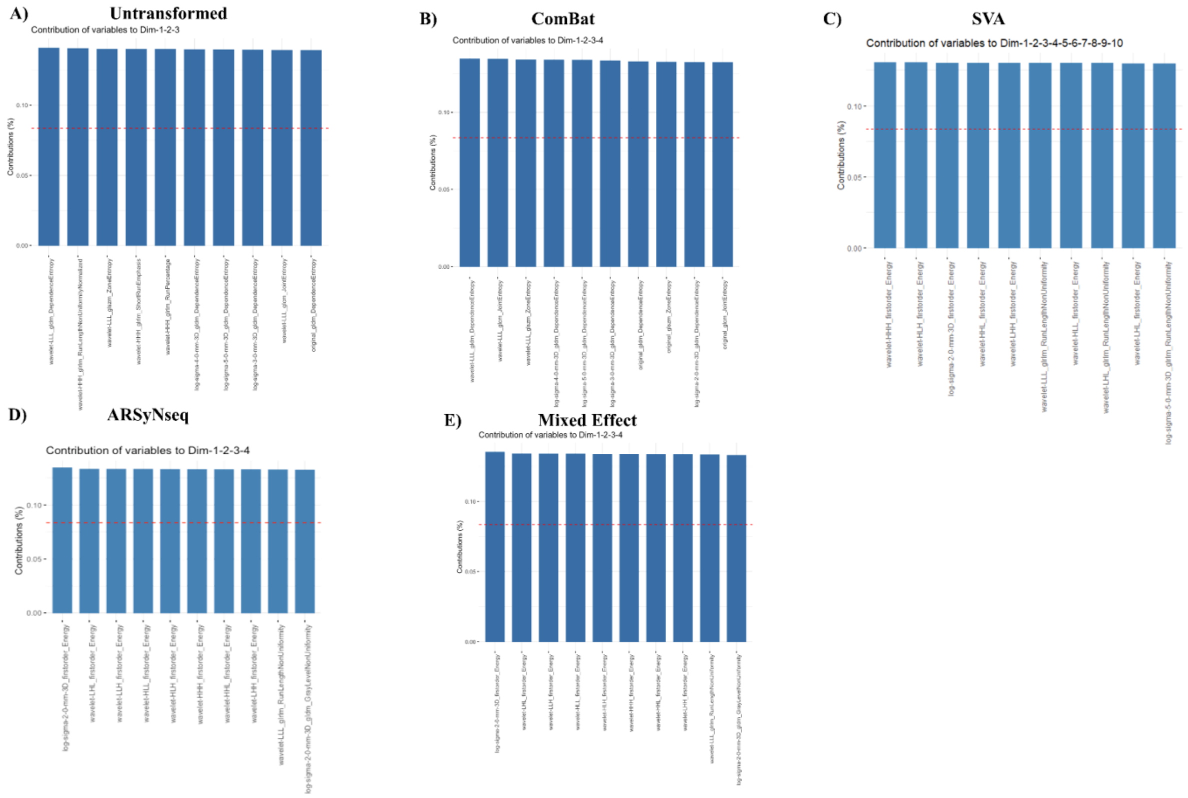

Supplementary Figure S1. Top 10 radiomic features that contributed to the PCs. (**A**) Untransformed only scaled; (**B**) ComBat; (**C**) SVA; (**D**) ARSyNseq and (**D**) Mixed effect model.
